# Assessment of references for the quantitative analysis of *LINE-1* and *Alu* methylation in cellular DNA and circulating cell-free DNA of cancer patients

**DOI:** 10.1371/journal.pone.0345087

**Published:** 2026-03-23

**Authors:** Tung The Pham, Linh Dieu Vuong, Tuan Van Mai, Son Van Ho, Giang Son Vu, Trang Thi Quynh Tran, Trang Hien Do, Oanh Minh Pham, Linh Thi Tu Nguyen, Loan Thi Phuong Pham, Lan Thi Thuong Vo, Uyen Quynh Nguyen

**Affiliations:** 1 Faculty of Biology, VNU University of Science, Hanoi, Vietnam; 2 Vietnam National Cancer Hospital, Hanoi, Vietnam; 3 175 Hospital, Ho Chi Minh City, Vietnam; 4 VNU Institute of Microbiology and Biotechnology, Hanoi, Vietnam; European Institute of Oncology, ITALY

## Abstract

The *LINE-1* and *Alu* retrotransposon elements, with more than 90% of their sequences being methylated, contribute to 30% of the human genome. Their hypomethylation profile, representing global methylation in cellular and cell-free DNA (cfDNA) from cancer, has been considered an attractive noninvasive biomarker of cancer. *LINE-1* and *Alu* methylation profiling has preferentially been performed by real-time methylation-specific PCR (qMSP), pyrosequencing, and methylation-sensitive high-resolution melting (MS-HRM), which are bisulfite-based PCR approaches that require reference sequences amplified by the Methylation Independent PCR (MIP) primers to normalize the quantification data. A technical weakness of MIP primers is unequal amplification, termed PCR amplification bias, leading to an under- or overestimation of expected methylation levels, and thus, hindering the effectiveness of DNA methylation-based biomarkers. To date, the PCR amplification bias of MIP primers that may affect the methylation analysis of repeat sequences such as *LINE-1* and *Alu* has not yet been described. Our study demonstrated for the first time the detrimental impact of biased MIP primers on *LINE-1* and *Alu* methylation profiles, causing a significant shift from the hypomethylated status to hypermethylated in cancer tissues and in cfDNA from cancer patients. Unexpectedly, this shift was also observed in cfDNA, even when quantified by the unbiased MIP primers, depending on the reference sequences. Our results suggest that an impartial reference for the methylation quantitation of repetitive elements, most importantly in cfDNA, should be further established to ensure cross-platform consistencies in DNA methylation profiling through bisulfite-based PCR techniques.

## Introduction

DNA methylation is one of the most crucial epigenomic mechanisms that governs the genome state and gene transcriptional programs [1]. Bisulfite conversion-based methods, the gold standard for quantifying and mapping methylation, which convert unmethylated cytosines to uracil residues while leaving methylated cytosines intact, have been massively utilized to profile the methylation status of whole genomes as well as local targets [2,3]. To date, the quantitative methylation-specific PCR (qMSP) method, a bisulfite-based method, is simple, highly sensitive, extremely cost-effective, and does not require any expensive infrastructure, and thus has been widely adopted not only in the research field but also in commercial tests [2,4]. DNA methylation levels quantified through qMSP are usually calculated by the relative comparative CT method (the ΔΔCT method), which requires methylation-specific PCR (MSP) primers derived from CpG-containing sequences to specifically amplify the methylated target, and methylation-independent PCR (MIP) primers, derived from the non-CpG-containing sequences as a reference to normalize qMSP data [3,5]. The MIP primers, used not only for the qMSP method but also for different bisulfite-based PCR methods, may preferably amplify the unmethylated alleles, leading to an unequal amplification termed PCR amplification bias, affecting the final estimates of DNA methylation and hindering the effectiveness of DNA methylation-based biomarkers [6–11]. Designing the MIP primers, either derived from the same or an unrelated locus as the MSP primers, based on the sequences of house-keeping genes that are completely unmethylated, has eliminated this amplification bias. However, house-keeping sequence-designed MIP primers are inappropriate for profiling the methylation of repeat sequences due to the improper relation between the copy number of single-copy genes (2 copies/genome) and that of repeat elements (>10^5^–10^6^ copies/genome) [12,13]. To date, several MIP primers have been widely used to quantify the DNA methylation level of repetitive sequences, although their PCR amplification bias has not yet been assessed.

The *LINE-1* and *Alu* retrotransposon elements, with more than 90% of their sequences being methylated, contribute to 30% of the human genome [12,14]. These repeat elements are particularly overrepresented in the cfDNA of healthy individuals and cancer patients [15,16]. Their hypomethylation profiles in cellular DNA and cell-free DNA (cfDNA) exhibit cancer-type signatures, thus, have been considered an attractive noninvasive biomarker of cancers [17,18]. However, the conflicting methylation levels of *LINE-1* and *Alu* observed in cellular and cfDNA, even when quantified in the same type of cancer or by an analytical approach, caused a disagreement about their clinical values [19–21]. This discrepancy in *LINE-1* and *Alu* methylation could be due to either the incomplete conversion by bisulfite [22,23], the biased amplification of the MIP primers [6,8–10], or the inappropriate references used for normalizing the qMSP data [13]. In addition, *LINE-1* elements are AT-rich sequences and enriched in the heterochromatin and gene-poor regions, while *Alu* elements are GC-rich sequences and enriched in the euchromatin and gene-rich regions [24]. Therefore, chromatin accessibility [24,25], endonuclease cleavages influenced by DNA methylation, fragmentations dependent on end-motif sites in these regions [26–28] and cytoplasmic synthesis via reverse transcription [29,30] are all involved in the different accumulation of the *LINE-1* and *Alu* fragments as well as their methylated/unmethylated proportions in cfDNA from various pathophysiological states [26]. Our previous report demonstrated a controversial hypermethylation of both these elements in tumour tissues and cfDNA from cancer patients compared to adjacent normal tissues and healthy individuals [23]. This disparity emphasized the need for further investigation into whether the MIP primers introduced any amplification bias and the appropriateness of the reference used for repeat sequences.

In this study, we evaluated the PCR amplification bias of different MIP primer pairs, including the pair used in our prior study [23]. We revealed that the biased MIP primers described in our previous study preferentially amplified the unmethylated allele, thus inaccurately profiling *LINE-1* and *Alu* hypermethylation. Other references amplified by unbiased MIP primers that are independent of local sequences revealed *LINE-1* and *Alu* hypomethylation in breast and lung tumor tissue samples when compared with adjacent normal tissue samples. This result is in line with the hallmark of *LINE-1* and *Alu* hypomethylation in cancer [31,32]. Unexpectedly, both *LINE-1* and *Alu*, while hypomethylated in cellular DNA, oscillated between the hypo- and hypermethylated status in cfDNA depending on the reference sequences, in other words, demonstrating a conflict with the originally hypomethylated status in cellular DNA observed in this study and previous studies [31–34], as well as with the cancer hallmark of *LINE-1* and *Alu* hypomethylation in cfDNA [18,32,35]. Our findings are the first to demonstrate the detrimental impact of using inappropriate references for the relative methylation quantification of repetitive sequences in cellular DNA, and more importantly, in cfDNA. Our findings also suggest a reassessment of the previously described global cfDNA methylation profile, which is contributed by several repeat elements and quantified by various references.

## Materials and methods

### Sample collection, DNA isolation, and bisulfite conversion

This study used DNA isolated from tumour tissues and their corresponding adjacent normal tissues from fresh-frozen biopsies of 146 primary breast cancer patients and 73 lung cancer patients, all of whom had been enrolled in our previous study [23]. Fifty-six paired samples were newly collected from lung cancer patients from November 1^st^ 2023 to April 30^th^ 2024. cfDNA was collected from blood samples of 247 healthy participants and 258 lung cancer patients, all of whom had also been enrolled in our prior study. Written informed consent was obtained from all patients and healthy participants prior to participation, and all sample collection procedures complied with relevant ethical guidelines and regulations by the Ethics Committee of the Vietnam Academy of Science and Technology (01–2023/NCHG-HDDD). Cellular DNA was extracted using the DNeasy Blood & Tissue Kit (Qiagen). cfDNA was extracted from 0.4 mL of plasma, collected from 1 mL of blood, using the MagMAX Cell-Free DNA Isolation kit (Thermo Fisher Scientific). A cellular DNA amount of 0.2 ng, quantified by the Qubit4 Fluorometer (Thermo Fisher Scientific), and 1 µL of cfDNA were respectively subjected to bisulfite conversion using the EZ DNA Methylation-Gold^®^ kit (Zymo Research). Two µL of bisulfite-treated DNA were used as a template for real-time PCR.

### Primer design

The MIP primer sets, including the previously described L1-Ref1 and the new L1-Ref, were designed to target non-CpG sequences located in the 5′ UTR region of the *H1LS* family (X58075). The MIP (Alu-Ref) primers were designed to target a non-CpG region in the *Alu* consensus sequences, following analyses on Repeat Masker and ClustalW [23,36]. The MSP primer sets (Me-L1, Me-Alu), described previously [23], were used in this study. Primer sequences, amplicon lengths, and qPCR conditions are presented in S1 Table in S1 File.

### Assessment of PCR amplification efficiency and PCR amplification bias

The PCR amplification efficiency was measured through standard curves built using the MIP primer sets and the MSP primer sets. Amplification efficiency was evaluated via (i) plotting the CT values against a serial dilution of bisulfite-treated human methylated DNA (Zymo Research) with amounts ranging from 1000 pg to 3.125 pg and (ii) plotting the ∆CT values obtained by subtracting the CT value of the MSP primer set from that of the MIP primer set, against the same serial dilution of DNA input. The standard curves allowed for selecting a CT cut-off value, a defined limit for the detection of a targeted amount in the sample. Any CT value above these defined limits would be considered false.

The PCR amplification bias was assessed by using 0.2 ng of control samples with defined methylation levels (0% − 100%), created by mixing fully methylated human DNA and fully unmethylated human DNA (Zymo Research). The control samples were bisulfite-treated and used as templates with either the MIP primer sets, specific to the reference sequences, or the MSP primer sets, specific to the methylated sequences. PCR amplification bias was measured by either (i) plotting the CT values from amplifying the bisulfite-converted control samples using the MIP primers against the methylation level of each control sample or (ii) plotting the DNA methylation level recovered after the qPCR reactions against the input DNA methylation level of each control sample [6].

### Quantification of *LINE-1* and *Alu* methylation

One µL of cfDNA and 0.2 ng of cellular DNA were converted by bisulfite. Two µL out of the eluted 20 µL were used as template for subsequent real-time PCR assays: (i) quantifying bisulfite-converted reference sequences using the MIP primers and (ii) quantifying the methylated *LINE-1* and *Alu* sequences using the MSP primers. A no-template control containing nuclease-free water was included in all PCR runs to monitor contamination. Real-time PCR was done with a volume of 20 µL per reaction using GoTaq qPCR Master Mix (Promega). All qPCR reactions were performed using the CFX96 Touch Real-Time PCR system (Bio-Rad).

### Methylation level calculation

Human methylated DNA (Zymo Research), defining the 100% methylation level, was used as the calibrator for the relative quantification of *LINE-1* and *Alu* methylation levels in samples [5,37]. Four reactions were carried out for each sample: two using the MSP primer sets to quantify the methylated *LINE-1* and *Alu* targets, and two others using the MIP primer sets for amplification of all bisulfite-converted reference sequences to normalize the qMSP data. Relative quantification of the methylated targets, detected using L1-Me and Alu-Me, regardless of the MIP primers, was calculated with the Livak and Pfaffl formulas, respectively [5,37]. Samples with both replicates returning a CT value greater than 30.25 for L1-Ref, and 25.55 for Alu*-*Ref were excluded from the study.

### Statistical analysis

In all dot plots, the ΔCT values are presented with the mean values and standard deviation, whereas *LINE-1* and *Alu* methylation levels are presented with the median values and interquartile range. Comparisons involving more than two groups with quantitative data were performed using the Kruskal-Wallis test. Comparisons between two groups with quantitative data were performed using the Unpaired T-test for independent samples, and the Paired T-test for pair-matched samples when normality was met, otherwise the Mann-Whitney U test for independent samples and the Wilcoxon matched-pairs signed rank test for pair-matched samples were used. Correlation between age and *LINE-1* and *Alu* methylation levels was assessed using Spearman’s rank correlation test. Simple linear regression was used to evaluate (i) the relationship between the DNA input and the recovered *LINE-1* and *Alu* methylation levels for PCR amplification bias assessment, and (ii) the relationship between the CT values and the DNA input. Statistical significance was defined as P < 0.05 for all statistical analyses. All statistical analyses and visualizations were performed using the GraphPad Prism® program version 10 (https://www.graphpad.com/scientific-software/prism/).

## Results

### Designing MIP primers specific to *LINE-1* and *Alu* elements

*LINE-1* and *Alu* elements are non-randomly fragmented and differently overrepresented in cfDNA depending on their methylation and the variant end-motifs presented on their sequences, which are altered in diseases [16,26,27]. Thus, we decidedly investigated their methylation in cfDNA using the references derived specifically from their own sequences. Two MIP primer sets, including the previously described L1-Ref1 and the new L1-Ref, were designed to target non-CpG sequences located in the 5′ UTR region of the *H1LS* family (X58075). The Alu-Ref primers were designed to target a non-CpG region in the *Alu* consensus sequences, following analyses on Repeat Masker [23,36] and ClustalW. The PCR amplification efficiency and PCR amplification bias were then assessed for all designed primers.

### Assessment of qPCR amplification efficiency

**Fig 1:**
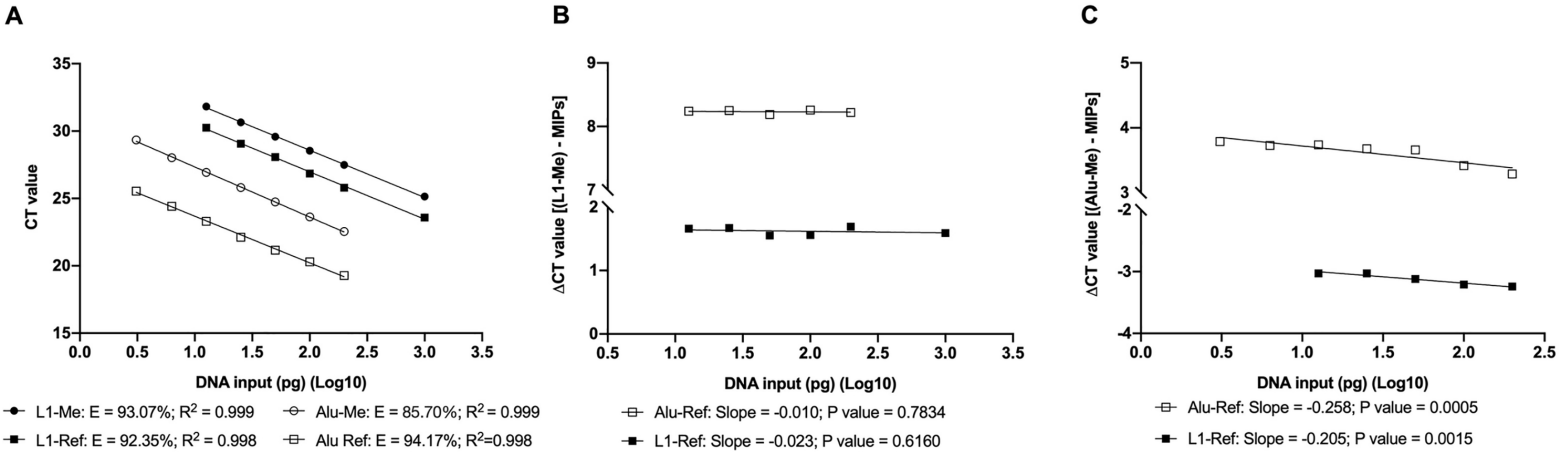
Evaluation of PCR amplification efficiency of the MIP and MSP primer sets on the fully methylated DNA control. (A) qPCR was performed using the MIP primer sets (L1-Ref and Alu-Ref) and the MSP primer sets (L1-Me, Alu-Me) on a bisulfite-treated serial dilution of human methylated DNA ranging from 1000 pg to 3.125 pg. The R^2^ values of all amplicons are greater than 0.99, the amplification efficiencies (E) are greater than 85%, indicating that the CT values of all primer sets have a strong linear relationship with the DNA input amount, and the qPCR conditions were optimized. (B, C). ∆CT values were calculated by subtracting the CT value of the MSP primer set from that of each MIP primer set for *LINE-1* (B) and *Alu* (C). For the Alu-Me primers, ∆CT values increased gradually as the input quantity decreased (slope absolute value > 0.1, P value < 0.05), indicating the Pfaffl formula is suitable for relative methylation quantification when using either MIP primer sets. On the contrary, for the L1-Me primers, ∆CT values remained unchanged (slope absolute value < 0.1, P value > 0.05), indicating the Livak formula is suitable for relative methylation quantification using either MIP primer sets. Simple linear regression was used in statistical analysis. The number of observations for each assay was ≥ 3.

The PCR amplification efficiency was assessed by constructing a standard curve using a serial dilution of bisulfite-treated methylated human DNA, with input amounts ranging from 1000 pg to 3.125 pg, and amplifying with either the MIP or the MSP primer sets. Standard curves obtained by plotting the CT values against the DNA input amounts revealed desired amplification efficiencies (85.70% − 94.17%) and a strong linear relationship between the CT value and the DNA templates for all primer sets tested (R^2^ > 0.99) (Fig 1A). Based on the obtained standard curves, the CT cut-off value, a maximum CT value necessary to amplify the targeted sequences up to a detectable level, was defined for the L1*-*Ref primer set (30.25) corresponding to an input of 12.5 pg, and for the Alu-Ref primer set (25.55) corresponding to an input of 3.125 pg, respectively (Fig 1A). The CT cut-off value is used as a proxy measure of the targeted amount in the sample, thus, any CT value above this defined limit would be considered false. Plotting the delta CT values of each pair of reference and the methylated target against the logarithm of the template input amount revealed that the *LINE-1* methylation level, normalized by L1-Ref and Alu-Ref, was suited to the Livak formula (Fig 1B), and the *Alu* methylation level, normalized by L1-Ref and Alu-Ref, was suited to the Pfaffl formula (Fig 1C) [6,37]. Both these formulas were then used to assess qPCR amplification bias of the three MIP primer sets L1-Ref1, L1-Ref, and Alu-Ref.

### Assessment of qPCR amplification bias of the designed MIP primers

Our previous study has demonstrated that a minuscule cellular DNA amount of 0.5 ng was enough for complete bisulfite conversion to profile *LINE-1* and *Alu* methylation [23]. The new primer sets designed in this study were used again to ensure that this DNA amount is appropriate for analyzing the methylation of these elements. Human methylated DNA (Zymo Research) amounts, corresponding to 50 ng, 5 ng, 1 ng, 0.2 ng, and 0.02 ng, were bisulfite converted and used as templates for qMSP with the MIP and MSP primer sets. A higher input amount of DNA correlated with a higher ΔCT value, which theoretically must be constant since fully methylated DNA (100%) was used (S1 Fig in S1 File). This result indicated a faulty underestimation of *LINE-1* and *Alu* methylation when the DNA input increased. The experimentally recovered *LINE-1* and *Alu* methylation levels were identical to the 100% methylation level when using less than 0.2 ng of DNA. We chose 0.2 ng of commercial human DNA for further investigation into the PCR amplification bias of the designed MIP primers.

**Fig 2:**
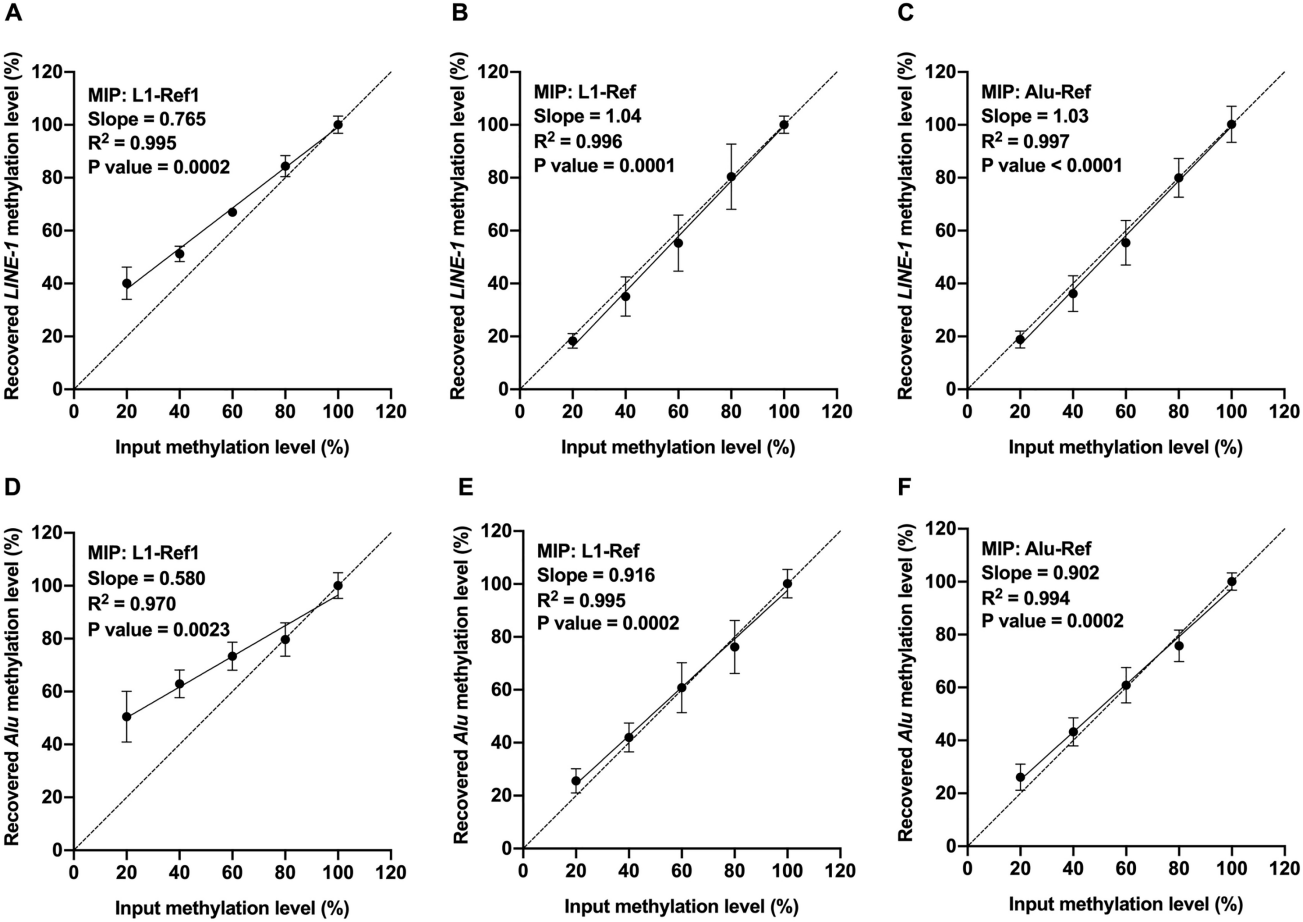
Evaluation of qPCR amplification bias. Control samples with defined methylation levels ranging from 0% to 100% were used as templates for qPCR. The formula appropriate for each primer set was applied to calculate the recovered methylation levels of *LINE-1* (A-C) and *Alu* (D-F). The recovered *LINE-1* and *Alu* methylation levels were much higher than the input methylation levels when using the L1-Ref1 primers (A, D), indicating that this primer set induced PCR amplification bias. However, when using the L1-Ref (B, E) and Alu-Ref primer sets (C, F), the recovered *LINE-1* and *Alu* methylation levels were nearly identical to the input methylation levels, revealing that these primer sets equally amplified all bisulfite-converted templates. Simple linear regression was used in statistical analysis. The number of observations for each assay was ≥ 4.

Fully methylated and fully unmethylated human DNA (Zymo Research) were mixed to create DNA control samples with methylation levels ranging from 0% to 100%. Two hundred pg of each control sample was bisulfite-treated and used as templates using three MIP primer sets specific to the reference sequences, and two MSP primer sets specific to the methylated targets. As shown in Fig 2, the experimentally recovered *LINE-1* methylation level of the control samples was identical to the input methylation levels when using the L1-Ref and Alu-Ref primers (Fig 2B, 2C), but not with the L1-Ref1 primers (Fig 2A). Similarly, the recovered *Alu* methylation level of the control samples was also identical to the input methylation levels solely when using the Alu-Ref and L1-Ref primer sets (Fig 2E, 2F). Taken together, these results indicate that the reference, amplified by the L1-Ref1 primers in our previous study, produced PCR amplification bias, but the references amplified by the L1-Ref and Alu-Ref primers did not, thus, the latter two references, hereafter referred to as *L1* and *Alu* references, were used for the assessment of *LINE-1* and *Alu* methylation levels in cfDNA.

### Methylation level analysis of *LINE-1* and *Alu* in the cfDNA of lung cancer patients and healthy individuals

**Fig 3:**
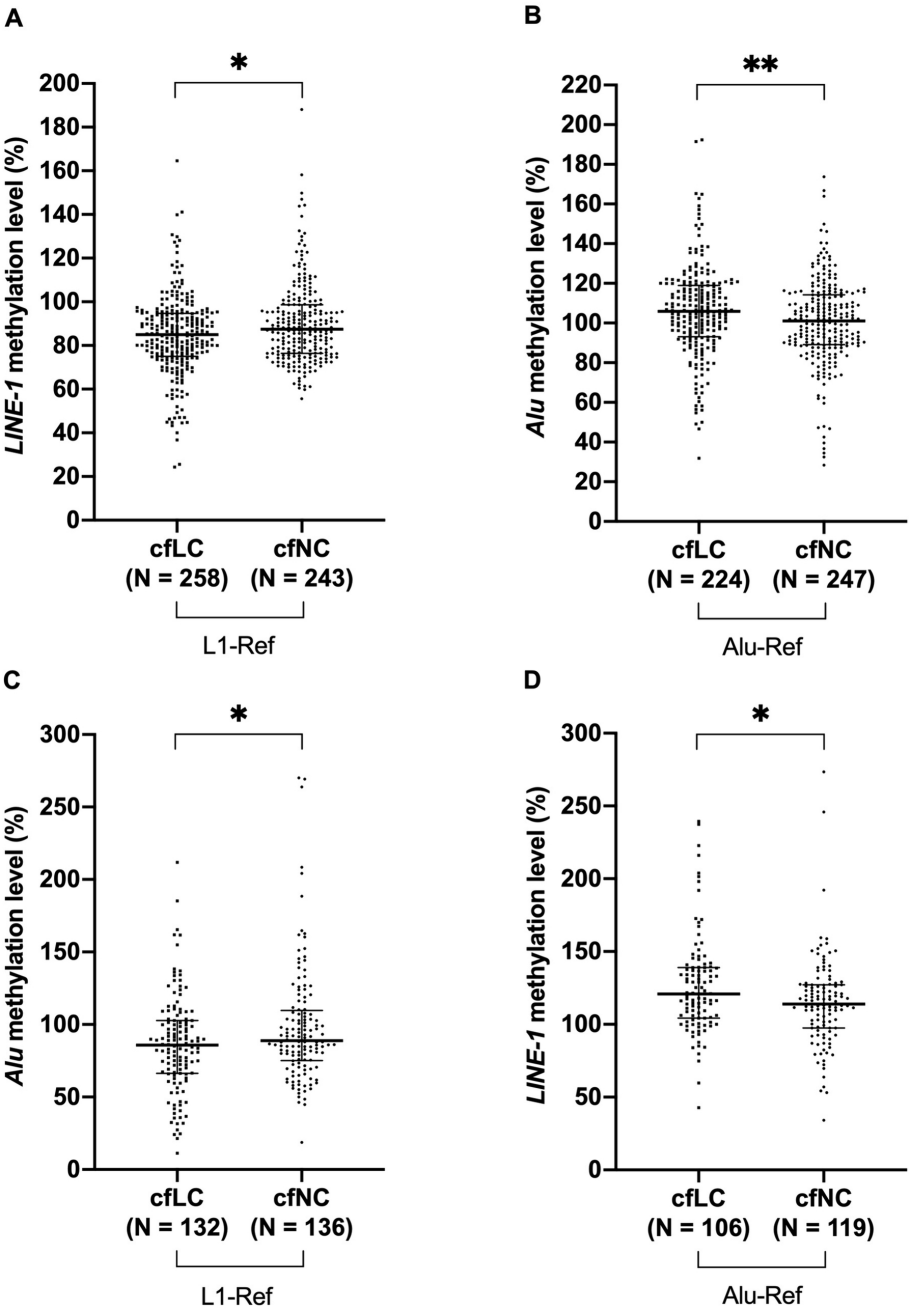
Methylation levels of *LINE-1* and *Alu* in cfDNA from lung cancer patients and healthy individuals. The methylation levels of *LINE-1* and *Alu*, normalized by either the *L1* reference (A, C) or the *Alu* reference (B, D), respectively decreased and increased in cfDNA from lung cancer patients (cfLC) as compared to that from healthy donors (cfNC). The Man-Whitney U test (A-D) was used in statistical analysis. (*) P < 0.05; (**) P < 0.01.

Two µL of bisulfite-converted cfDNA was quantitatively amplified with the MSP and MIP primer sets, deriving from both the *LINE-1* and *Alu* sequences, for analysis of their methylation level in the cfDNA samples extracted from 247 healthy donors and 258 primary lung cancer patients (Fig 3). Surprisingly, the methylation level of *LINE-1* normalized by the *L1* reference (Fig 3A), and that of *Alu* normalized by the *Alu* reference (Fig 3B) was of opposing trends; in other world, *LINE-1* methylation decreased (84.95% versus 87.50%) (P < 0.05) while *Alu* methylation increased (105.93% versus 101.07%) (P < 0.01) in cfDNA from lung cancer patients when compared with healthy individuals. Unexpectedly, when normalized by the same *L1* reference, both *LINE-1* and *Alu* methylation were significantly hypomethylated in cfDNA from lung cancer (84.95% and 85.88%, respectively) compared with healthy individuals (87.50% and 88.81%, respectively) (P < 0.05) (Fig 3A, 3C). However, when normalized by the *Alu* reference, both *LINE-1* and *Alu* were shown to be hypermethylated in lung cancer patients (120.93% and 105.93% versus 113.88% and 101.07%, respectively) (P < 0.05) (Fig 3B, 3D). The hypermethylated status of these elements in cfDNA from cancer patients, and the inversion of their methylation profiles depending on the reference MIP primers, are observations first reported by our study.

There were significant associations between the pathological stages of lung cancer patients and the *LINE-1* and *Alu* methylation levels in cfDNA when solely normalized by the *L1* reference (S2 Table in S1 File). In addition, the methylation level of *LINE-1*, but not of *Alu*, when normalized by the *L1* reference, correlated with ageing as *LINE-1* is more hypomethylated in older healthy individuals (P < 0.01) (S2 Fig in S1 File).

### Methylation level analysis of *LINE-1* and *Alu* in tumour and adjacent tissues

**Fig 4:**
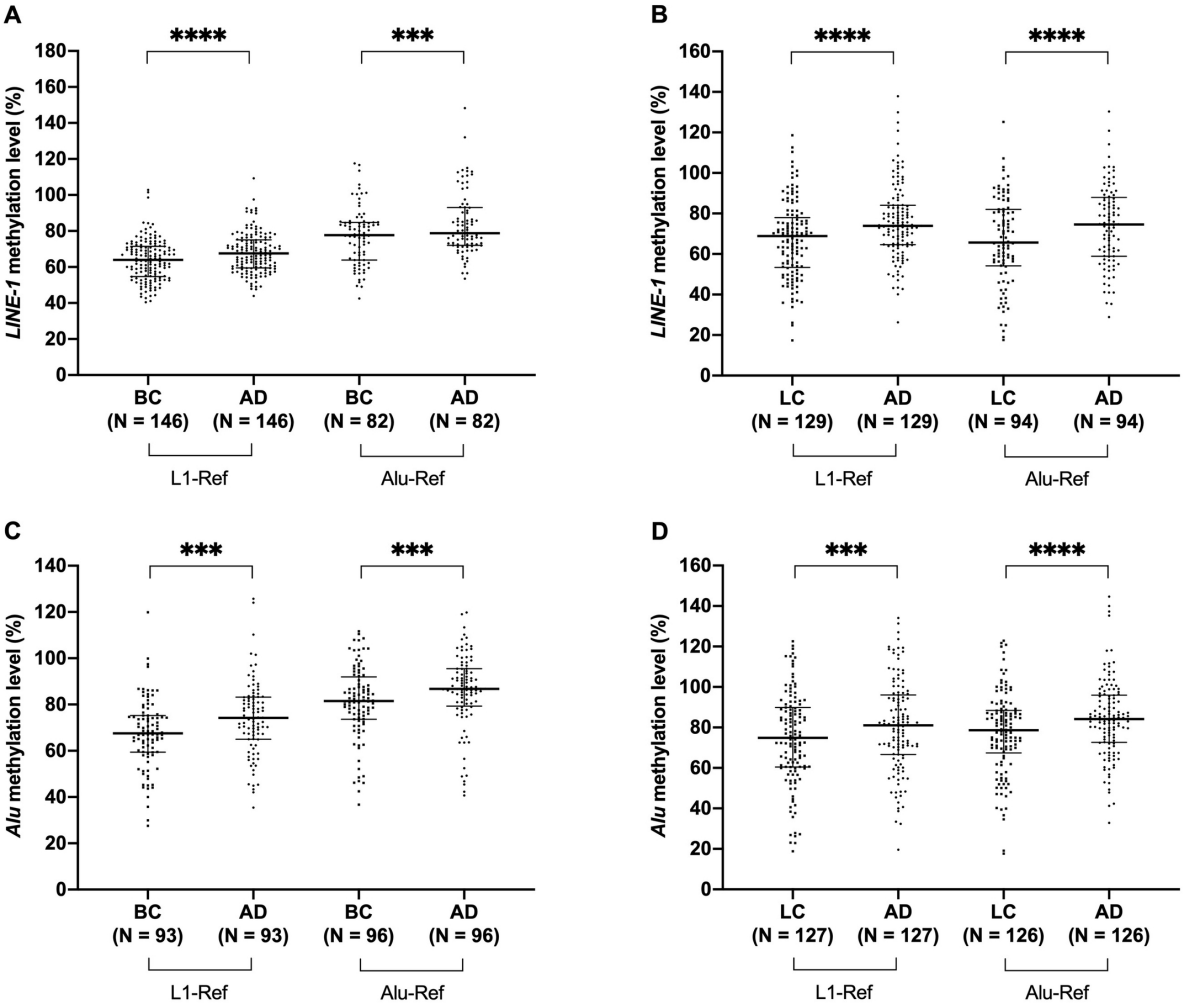
*LINE-1* and *Alu* methylation in tissues from cancer. Hypomethylation of *LINE-1* (A, B) and *Alu* (C, D) in breast tumour (BC) samples and in lung tumour (LC) samples as compared with their corresponding adjacent tissue samples (AD). Quantitative methylation data were normalized by either the *L1* or the *Alu* references. The Wilcoxon matched-pairs signed rank test (A-D), the Paired T-test (B-D) were used in statistical analysis. (***) P < 0.001; (****) P < 0.0001.

The inverse methylation status of *LINE-1* and *Alu* elements in cfDNA, depending on the references used to normalize, prompted us to assess their methylation state, with both MIP primer sets, in paired samples of tumour tissue and corresponding adjacent tissue collected from 146 primary breast cancer patients and 129 primary lung cancer patients. Significant hypomethylation of *LINE-1* and *Alu*, independent of the references, was observed in tumour samples compared to adjacent samples (Fig 4). In breast cancer patients (Fig 4A), *LINE-1* methylation levels calculated using the *L1* and *Alu* references in tumour samples (64.01% and 77.66%, respectively) significantly decreased compared to adjacent tissue samples (67.58% and 78.83%, respectively) (P < 0.001). Similarly, *LINE-1* methylation levels in lung tumour samples, normalized by both the *L1* and *Alu* references (68.88% and 66.37%, respectively), were significantly lower than that in adjacent tissue samples (73.91% and 74.02%, respectively) (P < 0.001) (Fig 4B). On the other hand, *Alu* also exhibited a decrease in methylation level in tumour tissues, regardless of the references (Fig 4C, 4D). When normalized by either the *L1* or *Alu* references, *Alu* methylation levels in breast tumour tissues (67.47% and 81.50%, respectively) and in lung tumour tissues (74.61% and 78.62%, respectively) were significantly decreased as compared to that in adjacent breast tissues (74.15% and 86.80%, respectively) and adjacent lung tissues (80.67% and 84.13%, respectively) (P < 0.001–0.0001) (Fig 4C, 4D).

There was no significant association between the clinicopathological characteristics of the breast and lung cancer tissues with *LINE-1* and *Alu* methylation levels when normalized by either reference, except for between the lung cancer subtype and *LINE-1* methylation when normalized by Alu-Ref (P = 0.0445) (S2 Table in S1 File).

## Discussion

The autonomous and active *LINE-1* and the non-autonomous *Alu* elements, contributing to around 30% of the human genome, are the most abundant repeated sequences in the human genome [14]. As both *LINE-1* and *Alu* are heavily methylated in normal cells and comprise a major portion of cfDNA, abnormal alterations in their methylation level and their fragmentomics in cfDNA make them an attractive biomarker of diseases and cancers [16,18,31]. *LINE-1* and *Alu* methylation profiling has preferentially been performed by bisulfite-based PCR techniques such as qMSP, pyrosequencing, and MS-HRM, which require references amplified by the MIP primers to normalize the quantification data [3,34]. Aside from the fundamental bisulfite-based techniques, high-throughput bisulfite-free techniques such as Enzymatic Methyl-seq (EM-seq) and Oxford Nanopore Technologies nanopore sequencing (ONT) have opened new opportunities for *LINE-1* and *Alu* methylation profiling and global DNA methylation detection. However, their application remains challenging due to the high level of laboratory expertise required, the discordant data on the methylation profile of repeated sequences, and the performance of DNA methylation calling tools that are still in the process of maturation [38,39]. Moreover, most cfDNA fragments are small in size (<200 bp), meaning the mapping of these sequences does not benefit from long-read sequencing instruments.

Relative quantification via references amplified by MIP primers has been preferentially used for DNA methylation profiling [2,40,41]. The MIP primers could be derived from the same or an unrelated locus as the methylated targets. For example, MIP primers derived from the single copy gene *ACTB* and the repetitive elements *Alu* have respectively been used for the methylation analysis of the *SEPT9* gene and the *Alu*, *LINE-1,* and *Sat2* elements [41,42]. A technical weakness of MIP primers is unequal amplification, leading to an under- or overestimation of expected methylation levels [6,7,9,11]. Thus, overcoming PCR amplification bias has been well recommended and supported by different mathematical pipelines for improving the performance of DNA methylation assays of specific single-copy targets [43–45]. In addition, using specifically designed MIP primers containing CpG dinucleotides to facilitate primer annealing to the methylated allele, and increasing the primer annealing temperature to inhibit the formation of secondary structures in GC-rich regions during PCR cycles, both have eliminated disproportional amplification [46,47]. Recently, reference materials and reference datasets have been developed for the calibration of technical biases and errors in methylation analysis of specific targets and whole genomes [8,48]. However, unequal amplification of the MIP primers used for relative methylation quantification of repeat sequences such as *LINE-1* and *Alu* has so far not been described.

Our study provides the first experimental evidence that PCR amplification bias can affect methylation quantification of the repetitive sequences *LINE-1* and *Alu*. PCR amplification of a serial dilution of control samples, with defined methylation levels ranging from 0% to 100%, revealed substantial deviations between the expected and observed *LINE-1* and *Alu* methylation when using the previously designed MIP primer set (Fig 2). The biased primers induced a shift towards the hypermethylated status due to the preferential amplification of unmethylated alleles, resulting in higher observed methylation levels as expected methylation decreased (Fig 2) and subsequently, a misinterpretation of *LINE-1* and *Alu* hypermethylation in cancer [23]. Using the unbiased MIP primer sets, derived from sequences either related or unrelated to the methylated targets, revealed *LINE-1* and *Alu* hypomethylation in tumour tissue samples compared to corresponding adjacent normal tissue samples (Fig 4), thus, in line with the prevailing hypomethylation hallmark of repeat elements in cancer [17,32,34].

The methylation status of *LINE-1* and *Alu* in cfDNA can reflect their methylation signatures in cellular DNA that was fragmented and released in the bloodstream through various mechanisms [33,49]. cfDNA fragmentation is a stepwise process involving chromatin accessibility, first by nuclear nucleases creating single- and double-stranded DNA of different lengths [25,50,51], then by cleavages by plasma nucleases whose activities are influenced by DNA methylation and the presence of end-motif sites within the sequences [26,27,52]. Euchromatin and gene-rich regions contain an abundance of *Alu* elements [53,54], and contribute as a main source of cfDNA [55], thus allowing for the over-representation of *Alu* fragments in cfDNA. cfDNA *Alu* contributes 43.53% of the total 100 bp fragments among the main repetitive elements in cfDNA, while cfDNA *LINE-1* makes up only 5.88% [16]. The difference in the number of 5’ UTR *LINE-1* sequence present in cfDNA from lung cancer patients was insignificant compared to that from healthy individuals [23]. Most somatic *LINE-1* insertions are heavily 5’UTR-truncated, likely explained by the host cleavage of *LINE-1* RNA before reverse transcription [56,57], thus explaining the insignificant variance of the 5’UTR *LINE-1* copy numbers in cancer. For instance, there exist only 34 full-length *LINE-1* among 1,708 somatic *LINE-1* retrotransposition events that were detected in colorectal epithelium [58]. On the contrary, the number of short-sized and long-sized *Alu* fragments present in cfDNA was shown to have opposing changes in lung cancer patients when compared to healthy individuals [23,35]. The chaotic expression of *Alu*-containing genes at increased heterogeneity [59], together with the specific-G quadruplex-structure of *Alu* sequences that influences not only short double-stranded but also single-stranded cfDNA fragmentation [50,51,60], can all contribute to the abnormality in *Alu* fragment size and abundance released into cfDNA from cancer cells. For this reason, it is plausible that the *Alu*-derived reference influenced the methylation quantification of repeat elements, leading to the observed hypermethylation of *LINE-1* and *Alu* in cancer cfDNA, which differs from the hypomethylation hallmark observed in cellular DNA. Indeed, most studies investigating *LINE-1* and *Alu* methylation in cfDNA using the gold standard method of bisulfite-based sequencing [18,28,31,35] or bisulfite-free methods such as absolute quantitative analysis of methylated alleles (AQAMA) assay, QUAlu (Quantification of Unmethylated *Alu*), and methylation-sensitive restriction enzyme digestion and qPCR assay [61–63] have all reported *LINE-1* and *Alu* hypomethylation across different cancer types when compared with healthy individuals. Our study, when using the unbiased and appropriate *LINE-1* reference derived from the 5’ UTR *LINE-1* sequence, also consistently described *LINE-1* and *Alu* hypomethylated status in cfDNA from lung cancer patients. Altogether, changes in cfDNA methylation profiles, most extensively quantified by the qMSP method [18], as was done in our study, should be further evaluated in order to better understand the methylation patterns of repetitive sequences in cfDNA.

## Conclusion

The novelty of our result is the emphasis that a choice of inappropriate MIP primers that can induce PCR bias or amplify targets of complex structure and altered copy number for data normalization may result in a fundamental reversal of methylation profile not only in cellular DNA but also in cfDNA, thus leading to a misinterpretation of biological effects.

This study has demonstrated inconsistencies in *LINE-1* and *Alu* methylation profiling arising from technical biases and analytical protocols. *LINE-1* and *Alu* elements are major contributors to cfDNA and reflect the state of global methylation, thus making their methylation analysis a preferred non-invasive approach, particularly for personalized applications. An appropriate reference for the methylation quantification of repetitive sequences, especially in cfDNA, should be further established to ensure cross-platform consistencies in the methylation profiling of repetitive elements through bisulfite-based PCR techniques.

## Supporting information

S1 File **S1 Table**. Primer sets and quantitative real-time PCR conditions for the quantification of *LINE-1* and *Alu* methylation levels. The methylation-dependent-specific PCR (MSP) primers (Me-) and the methylation-independent PCR (MIP) primers were designed based on the consensus sequences of *LINE-1* and *Alu* [23]. All non-CpG cytosines have been replaced by “t” in the forward primers and by “a” in the reverse primers. **S2 Table**. Correlation between the clinicopathological characteristics of cancer patients and *LINE-1* and *Alu* methylation status in tissues of breast cancer and lung cancer (A) and in cfDNA from lung cancer patients (B). **S1 Fig**. The effect of excessive DNA input for bisulfite conversion on PCR amplification efficiency and the methylation levels of *LINE-1* and *Alu*. The amplification efficiency of the MIP primers and MSP primers to different amounts of fully methylated human DNA (50 ng, 5 ng, 1 ng, 0.2 ng, and 0.02 ng). The ΔCT values significantly differ at input amounts from 50 ng to 1 ng with the *LINE-1* (A) and *Alu* (B) targets. An increase in DNA input for bisulfite conversion was shown to associate with an under-methylated level of *LINE-1* (C) and *Alu* (D). The Unpaired t-test was used in statistical analysis. The number of observations for each assay was ≥ 4. (ns) P > 0.05; (**) P < 0.01; (****) P < 0.0001. **S2 Fig**. Correlation between age and *LINE-1* and *Alu* methylation levels. *LINE-1* methylation levels in cfDNA correlated with the age of healthy individuals (A) but did not with the age of lung cancer patients (B). Alu methylation levels in cfDNA are uncorrelated with the age of healthy individuals (C) and lung cancer patients (D). Methylation assessments were performed on two microliters of bisulfite-converted cfDNA. Spearman’s rank correlation test (A – D) was used in statistical analysis.
(ZIP)
