# Supplementary material for: Assessment of references for the quantitative analysis of LINE-1 and Alu methylation in cellular DNA and circulating cell-free DNA of cancer patients
Source: PLoS One. 2026 Mar 23;21(3):e0345087. doi: 10.1371/journal.pone.0345087 (PMC13008044; doi:10.1371/journal.pone.0345087)
Supplement: S1 File — S1 Table. Primer sets and quantitative real-time PCR conditions for the quantification of LINE-1 and Alu methylation levels. The methylation-dependent-specific PCR (MSP) primers (Me-) and the methylation-independent PCR (MIP) primers were designed based on the consensus sequences of LINE-1 and Alu [23]. All non-CpG cytosines have been replaced by “t” in the forward primers and by “a” in the reverse primers. S2 Table. Correlation between the clinicopathological characteristics of cancer patients and LINE-1 and Alu methylation status in tissues of breast cancer and lung cancer (A) and in cfDNA from lung cancer patients (B). S1 Fig. The effect of excessive DNA input for bisulfite conversion on PCR amplification efficiency and the methylation levels of LINE-1 and Alu. The amplification efficiency of the MIP primers and MSP primers to different amounts of fully methylated human DNA (50 ng, 5 ng, 1 ng, 0.2 ng, and 0.02 ng). The ΔCT values significantly differ at input amounts from 50 ng to 1 ng with the LINE-1 (A) and Alu (B) targets. An increase in DNA input for bisulfite conversion was shown to associate with an under-methylated level of LINE-1 (C) and Alu (D). The Unpaired t-test was used in statistical analysis. The number of observations for each assay was ≥ 4. (ns) P > 0.05; (**) P < 0.01; (****) P < 0.0001. S2 Fig. Correlation between age and LINE-1 and Alu methylation levels. LINE-1 methylation levels in cfDNA correlated with the age of healthy individuals (A) but did not with the age of lung cancer patients (B). Alu methylation levels in cfDNA are uncorrelated with the age of healthy individuals (C) and lung cancer patients (D). Methylation assessments were performed on two microliters of bisulfite-converted cfDNA. Spearman’s rank correlation test (A – D) was used in statistical analysis. (ZIP) [file pone.0345087.s001.zip › S2 Fig.-Pham et al-PLoS ONE.pdf]

Assessment of references for the quantitative analysis of *LINE-1* and *Alu* methylation in cellular DNA and circulating cell-free DNA of cancer patients

Tung The Pham[1¶], Linh Dieu Vuong[2¶], Tuan Van Mai [2], Son Van Ho[3], Giang Son Vu[3], Trang Thi Quynh Tran[1], Trang Hien Do[1], Oanh Minh Pham[1], Linh Thi Tu Nguyen[1], Loan Thi Phuong Pham[1], Lan Thi Thuong Vo[1,4*] Uyen Quynh Nguyen[4*]

[1] Faculty of Biology, VNU University of Science, Hanoi. [2] Vietnam National Cancer Hospital. [3] 175 Hospital, Ho Chi Minh City, Vietnam. [4] VNU Institute of Microbiology and Biotechnology.

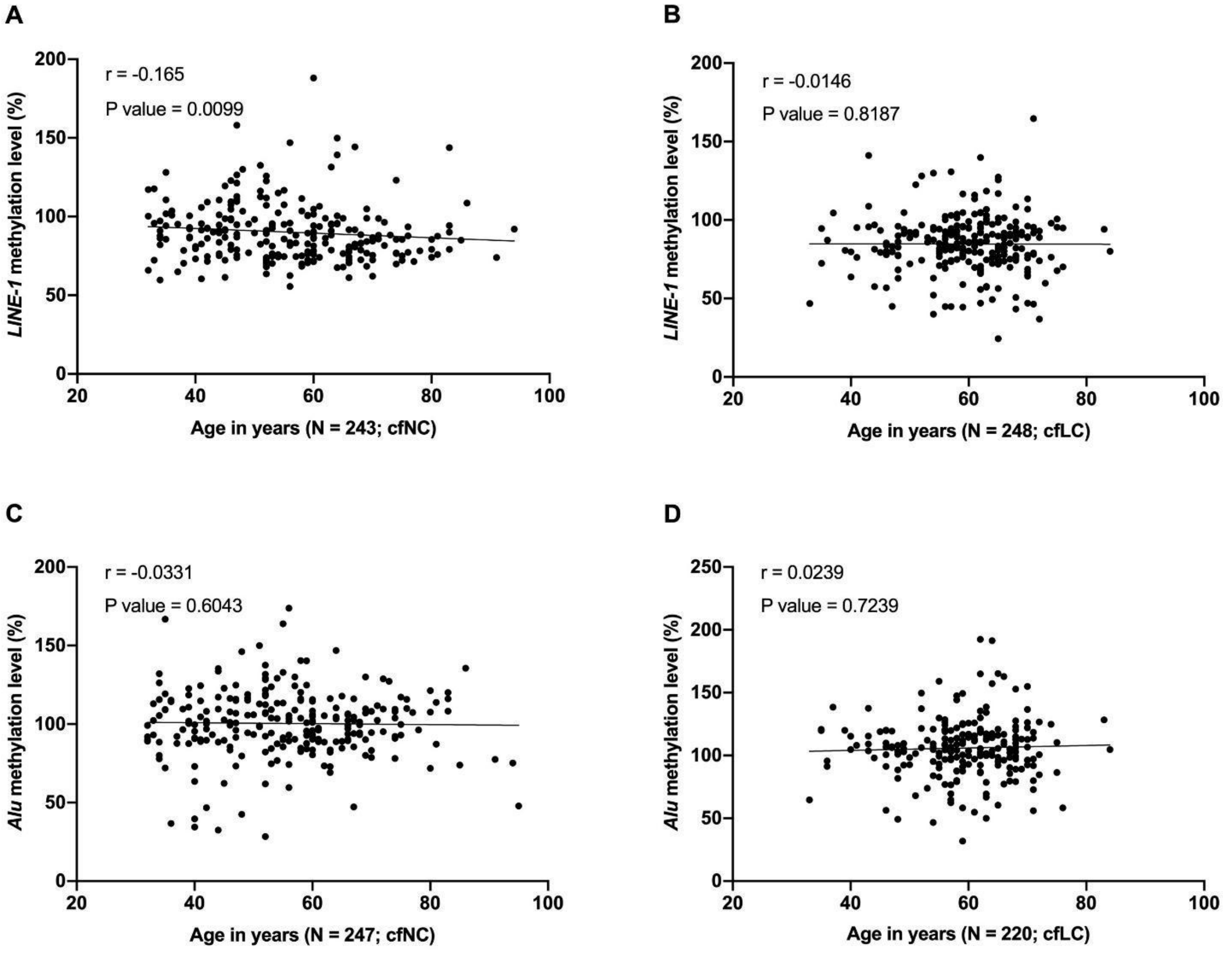

S2 Fig. Correlation between age and *LINE-1* and *Alu* methylation levels. *LINE-1* methylation levels in cfDNA correlated with the age of healthy individuals (A) but did not with the age of lung cancer patients (B). *Alu* methylation levels in cfDNA are uncorrelated with the age of healthy individuals (C) and lung cancer patients (D). Methylation assessments were performed on two microliters of bisulfite-converted cfDNA. Spearman's rank correlation test (A - D) was used in statistical analysis.
